# Red Ginseng Extract Intake and Changes in Metabolite Profiles, Gut Microbiota, and Immune Responses of Healthy Rats

**DOI:** 10.3390/nu18091462

**Published:** 2026-05-02

**Authors:** Madhuri Sangar, Seong-Hwa Song, Saoraya Chanmuang, Dong-Shin Kim, Gwang-Ju Jang, Hyeon-Jeong Lee, Young Kyoung Rhee, Hee-Do Hong, Chang-Won Cho, Hyun-Jin Kim

**Affiliations:** 1 Division of Applied Life Sciences (BK21 Four), Gyeongsang National University, 501 Jinjudae-ro, Jinju 52828, Republic of Korea; madhurysangar@gmail.com (M.S.);; 2 Department of Food Science & Technology, Institute of Agriculture and Life Science, Gyeongsang National University, 501 Jinjudae-ro, Jinju 52828, Republic of Korea; 3 Korea Food Research Institute, 245 Nongsaengmyeong-ro, Wanju-gun 55365, Republic of Korea; ykrhee@kfri.re.kr (Y.K.R.); cwcho@kfri.re.kr (C.-W.C.); 4 Department of Toxicology and Drug Chemistry, Defense Forensics Service, Criminal Investigation Command, 22 Itaewon-ro, Seoul 04383, Republic of Korea

**Keywords:** gut microbiota, immune, metabolomics, red ginseng, carbohydrates

## Abstract

**Background:** Red ginseng (RG) exhibits enhanced bioactivity compared to white ginseng. Although the beneficial effects of RG have been well investigated in disease models, its impacts on the metabolome, gut microbiota, and immune response under normal physiological conditions remain poorly understood. **Methods:** Rats were randomized into three groups: control (normal diet), RL (low-dose RGE at 100 mg/kg body weight), and RH (high-dose RGE at 200 mg/kg body weight). After five weeks, metabolite profiles of the blood, liver, kidney, and large intestinal contents were analyzed and the gut microbiota was assessed. Splenocytes were isolated and treated with or without ethanol-precipitated carbohydrate fractions isolated from RGE or from intestinal contents, and IL-12 secretion was measured. Additionally, the correlations among biochemical characteristics, metabolites, gut microbiota, and immune markers were analyzed. **Results:** RGE intake decreased plasma triglycerides, liver function biomarkers, and epididymal adipose tissue weight. It also altered metabolite profiles for plasma, liver, kidney, and intestinal contents and increased the hepatic NAD^+^/NADH ratio. RGE intake reduced the populations of harmful bacteria, whereas it increased *Lachnospiraceae*. RGE intake enhanced IL-12 production in splenocytes. Furthermore, splenocytes treated with carbohydrates isolated from the small and large intestinal contents of RGE-fed rats secreted higher IL-12 levels than those of the control group. **Conclusions:** RGE modulated the gut microbiota, metabolism, and immune responses in healthy rats under normal physiological conditions, warranting further investigation into the underlying mechanisms.

## 1. Introduction

Ginseng (*Panax ginseng* Meyer) is widely used as a dietary supplement due to its various potential health-promoting properties. Numerous animal and clinical studies have demonstrated that ginseng-derived bioactive compounds such as ginsenosides, polyphenols, flavonoids, polysaccharides, and vitamins have protective effects against tumors, cancer, oxidative stress, and diabetes, as well as enhancing immune function [1,2,3,4,5]. Red ginseng (RG), produced by steaming and drying ginseng roots, exhibits superior pharmacological efficacy compared to white ginseng (WG). The steaming process alters the chemical composition of ginseng, thereby enhancing its biological activity [6]. In particular, the levels of ginsenosides such as Rg1, Re, Rb1, Rc, R2, Rb3, Rd, and notoginsenoside R1 are reduced after steaming ginseng at 120 °C for 2–4 h, while increasing the concentrations of ginsenosides like Rh1, Rg2, 20R-Rg2, Rg3, Rg5, and Rh2. RG has demonstrated greater antiasthmatic effects in mice and enhanced antiproliferative activity in human breast cancer cells compared to WG [7,8]. In addition, RG has been extensively studied in clinical trials, including placebo-controlled studies, and in animal and biochemical studies, focusing on its impacts on hypertension [9], renal failure [10], and apoptosis [11] across various disease models [12,13]. However, the pharmacological effects of ginseng can be influenced by its distribution, absorption, metabolism, and subsequent excretion processes, which may involve both liver metabolism and interactions with gut bacteria [14].

Among these, the gut microbiota plays a significant role in the effectiveness of ginsenosides and other bioactive compounds of ginseng as they undergo modifications within the gastrointestinal (GI) tract after consumption, which make them significant determinants of host health and disease [15]. Ginseng components like Rb1 and Rb2 are metabolized into hydrophobic forms in the stomach and gut with the help of gastric juice and the microbiota. Moreover, gut bacteria influence host physiology through their metabolic activity, affecting immunity, inflammation, neurophysiology, and metabolism, as well as disease progression [16]. In recent studies, multi-omics technologies were implemented for a better understanding of the impacts of RG intake on the gut microbiota and physiological characteristics. However, most of the existing studies on ginseng have primarily focused on its therapeutic effects in disease models, rather than its effects under normal physiological conditions. While this approach provides valuable insights, it does not accurately represent the common consumption of RG among healthy individuals seeking to maintain or enhance their overall health. The effects of RG under normal physiological conditions remain largely understudied.

In this study, a comprehensive analysis was conducted to investigate the effects of red ginseng extract (RGE) intake on metabolites, gut microbiota, and immune function in healthy rats and to examine the correlations among these parameters.

## 2. Materials and Methods

### 2.1. Red Ginseng Extract (RGE)

The red ginseng used in this study was purchased from Kuan Industry (Seoul, Republic of Korea), extracted with water under reflux at 100 °C for 4 h, and concentrated using a rotary evaporator. Solid content was determined gravimetrically, total sugars by the phenol–sulfuric acid method, acidic polysaccharides by the carbazole–sulfuric acid method, and total phenolic compounds by the Folin–Ciocalteu method. The resulting RGE had final solid content of 480.50 mg/g, including 70.07% total sugars, 26.84% acidic polysaccharides, and 0.78% total phenolic compounds (Table S1). The content of individual ginsenosides, expressed as mg/100 mL of extract, was as follows: 101.1 mg of Rb1, 112.4 mg of Rb2, 21.6 mg of Rb3, 202.4 mg of Rc, 60.3 mg of Rd, 161.5 mg of Rg3, 94.1 mg of Re, 87.3 mg of Rg1, 44.7 mg of Rg2 + Rh1, and 2.6 mg of Rh1.

### 2.2. Animals and Red Ginseng Administration

The animal study was approved by the Animal Research Committee of Gyeongsang National University (Permit Number: GNU-140905-R0040). Four-week-old male Sprague- Dawley rats were purchased from Koateck (Pyeongtaek, Republic of Korea) and housed under controlled conditions with a 12 h light–dark cycle at room temperature (22 ± 2 °C) and humidity of 55%. The rats had free access to a commercial diet and tap water. After a two-week adaptation period, the rats were divided into three groups: control (Con, n = 8), low-dose RGE (RL, 100 mg RGE/kg body weight, n = 10), and high-dose RGE (RH, 200 mg RGE/kg body weight, n = 10). The RL and RH groups received RGE orally for five weeks, while the control group received water. Food intake and body weight were recorded weekly. Upon sacrifice, the rats were anesthetized with diethyl ether, and blood was collected from the postcaval vein. The liver, kidney, epididymal fat, small intestinal contents (SIC), and large intestinal contents (LIC) were collected, frozen in liquid nitrogen, and stored at −80 °C until analysis. The sample size was determined based on previous experience with similar animal studies and practical considerations; no formal statistical power calculation was performed before the experiment. Healthy male Sprague-Dawley rats that completed the acclimatization period and showed no abnormal clinical signs were included in the study, and no animals were excluded after allocation. For biochemical parameters, only five samples per group were analyzed because the available blood volume was insufficient for all assays.

### 2.3. Biochemical Characteristics

Triglycerides (TG), total cholesterol (TC), low-density lipoprotein (LDL) cholesterol, high-density lipoprotein (HDL) cholesterol, alanine aminotransferase (ALT), and aspartate aminotransferase (AST) in plasma were measured using commercial assay kits (Roche Diagnostics, Basel, Switzerland). The NAD^+^/NADH ratio in the liver was quantified using a NAD^+^/NADH quantification kit (Sigma-Aldrich, St. Louis, MO, USA). The quantification of nitrate and nitrite in plasma and liver tissue was performed using a nitrite/nitrate assay kit (Sigma-Aldrich).

### 2.4. Gut Microbiota Analysis

Fecal bacterial DNA was extracted using the QIAamp DNA Stool Mini Kit (Qiagen, Hilden, Germany) [17]. The V1–V2 region of 16S rDNA was amplified using primers 8F and 338R on a FastStart High Fidelity System (Roche Diagnostics, Basel, Switzerland) under the following conditions: 94 °C for 3 min; 35 cycles of 94 °C for 15 s, 55 °C for 45 s, 72 °C for 1 min, and 72 °C for 8 min for a final extension. Amplicons were purified using AMPure beads (Beckman Coulter, Brea, CA, USA) and libraries prepared using the Ion Xpress Plus Fragment Library Kit (Thermo Scientific, Wilmington, DE, USA). Library quality was assessed on the Bioanalyzer 2100 (Agilent Technologies, Inc., Santa Clara, CA, USA) using high-sensitivity DNA chips. Sequencing was performed on an Ion Torrent PGM system (Thermo Scientific). Data were processed using QIIME2 version 2020.08 against the SILVA database and analyzed using Mothur version 1.44.3.

### 2.5. Metabolomic Analysis

#### 2.5.1. Sample Preparation for Metabolomic Analysis

MS-based metabolomics analysis was performed using a previous method [18] with minor modifications. Metabolites were extracted from plasma and lyophilized liver, kidney, and LIC. Plasma was mixed with cold acetone (1:1, *v*/*v*) to precipitate proteins, while lyophilized samples were homogenized with cold acetonitrile (ACN). After centrifugation, the supernatants were completely dried. For ultra-performance liquid chromatography–quadrupole time-of-flight mass spectrometry (UPLC-Q-TOF MS) analysis, the residues were resolved with 20% methanol containing terfenadine as an internal standard (IS). For gas chromatography–mass spectrometry (GC-MS) analysis, dried samples were dissolved in methoxyamine hydrochloride with pyridine (20 mg/mL) containing dicyclohexyl phthalate as an IS, incubated at 37 °C for 90 min, and derivatized with N,O-bis(trimethylsilyl)trifluoroacetamide (BSTFA) containing 1% trimethylchlorosilane at 70 °C for 30 min.

#### 2.5.2. UPLC-Q-TOF MS Analysis

UPLC-Q-TOF MS analysis was carried out on an Acquity UPLC-Q-TOF device equipped with an Acquity UPLC BEH C18 column (2.1 mm × 100 mm, 1.7 µm; Waters, Milford, MA, USA). Mobile phases were 0.1% formic acid in water (A) and ACN (B) at 0.35 mL/min. The eluents were detected with a Q-TOF MS device in positive electrospray ionization (ESI) mode under optimized conditions: a desolvation gas flow rate of 800 L/h, a desolvation temperature of 400 °C, a source temperature of 120 °C, a capillary voltage of 3 kV, and a sampling cone voltage of 40 V. Leucine-enkephalin was used as a lack mass. MS data were processed in MassLynx software version 4.2 (Waters), aligned, and normalized to the IS. The metabolites were tentatively identified using the online database connected to UNIFI version 1.9.2 (Waters) [19].

#### 2.5.3. GC-MS Analysis

GC-MS analysis was performed on a GC-2010 plus device (Shimadzu, Tokyo, Japan) equipped with a DB-5 capillary column (30 m × 0.25 mm, 0.25 µm, Agilent) and a GCMS-TQ 8030 MS device (Shimadzu). Helium was used as the carrier gas at 1 mL/min. The injector temperature was 200 °C. The oven temperature gradient program was as follows: maintained at 70 °C for 2 min; increased to 150 °C at 5 °C/min, 210 °C at 3 °C/min, and 320 °C at 8 °C/min; and held at 320 °C for 8 min. The eluents were ionized in electron ionization mode (70 eV) on a GC-MS-TQ 8030 MS device with a source temperature of 230 °C and an interface temperature of 280 °C. Peaks were aligned and identified using the Wiley and NIST mass spectral databases, and retention indices (RIs) were calculated using a series of C8-C40 *n*-alkanes [19].

### 2.6. Analysis of Short-Chain Fatty Acids (SCFAs)

As previously described [20], SCFAs were quantified and determined using GC-MS, with minor modifications. SCFAs in the LIC samples were extracted from 30 mg of sample by mixing with 300 µL of hexane. After centrifugation, 200 µL of the supernatant was mixed with 100 µL of distilled water, 10 µL of HCl, and 400 µL of ether. After shaking for 5 min, the ether layer was transferred to a GC vial and derivatized with 20 µL of BSTFA at 70 °C for 20 min and then incubated at 37 °C for 2 h. The derivatized samples were analyzed using GC-MS using the same settings as above, with a modified oven program: 100 °C for 0.5 min, ramp to 180 °C at 8 °C/min (hold 1 min), ramp to 200 °C at 20 °C/min, and hold at 200 °C for 5 min. SCFAs were quantitatively analyzed based on authentic standards.

### 2.7. Measurement of IL-12 in Spleen Cells and IgA in Serum

The spleens were collected from rats fed a diet with or without RGE; washed with RPMI 1640 medium containing 10% FBS, 1% penicillin–streptomycin, and 50 µM 2-mercaptoethanol; and gently dissociated through a 70 μm cell strainer. After red blood cell lysis, splenocytes were seeded at 1 × 10^6^ cells/well in 48-well plates and cultured in RPMI 1640 medium at 37 °C with 5% CO_2_ for 72 h. The culture supernatants were stored at −80 °C for IL-12 analysis. Carbohydrate fractions were obtained from RGE and from the dried intestinal contents (SIC and LIC) of the control, RL, and RH groups with 90% aqueous ethanol. After centrifugation, the pellets were resuspended in distilled water. The splenocytes from control rats were cultured using the same culture conditions with or without the carbohydrate fractions. After 72 h, IL-12 was measured using IL-12 ELISA kits (Invitrogen, Camarillo, CA, USA). IgA in serum was determined using an IgA ELISA kit (Invitrogen).

### 2.8. Statistical Analysis

The processed LC-MS and GC-MS data sets were statistically analyzed by multivariate statistical analysis using SIMCA-P+ version 12.0.1 (Umetrics, Umeå, Sweden). The biological characteristics of the rats and the normalized intensities of the metabolites were analyzed using one-way analysis of variance (ANOVA) with Duncan’s test (*p* < 0.05) in SPSS 17.0 (SPSS Inc., Chicago, IL, USA). Statistical differences in the gut microbiota composition were determined by the Kruskal–Wallis test. The correlation analysis was performed using R software version 4.3.2.

## 3. Results

### 3.1. Biochemical Characteristics

The biochemical characteristics of the rats in the control, RL (100 mg/kg), and RH (200 mg/kg) groups indicated that RGE intake significantly decreased the blood TG, ALT, and AST levels by 33%, 13%, and 24%, respectively, compared to the control group, while the LDL level was increased by 31%. Adipose tissue was also significantly reduced in the RH group. However, body weight, food intake, liver weight, and the levels of TC and HDL were not significantly affected by RGE intake (Table 1).

### 3.2. Gut Microbiota

**Figure 1:**
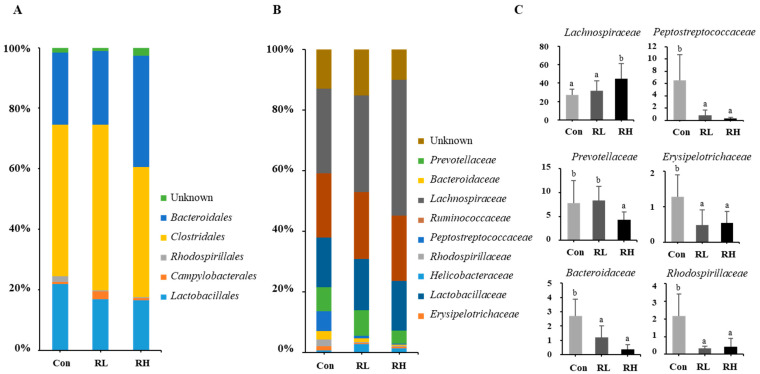
Effects of red ginseng extract (RGE) intake on gut microbiota composition at the (**A**) phylum level and (**B**) family level and (**C**) the relative abundances of major bacterial families. Control; RL, low-dose RGE (100 mg RGE/kg body weight); RH, high-dose RGE (200 mg RGE/kg body weight). Data represented as mean ± SD. Bars with different letters are significantly different (*p* < 0.05).

RGE intake altered the gut microbiota composition in rats (Figure 1A–C). The rarefaction curves reached a saturation plateau, indicating sufficient sampling depth (Figure S4). At the order level, RGE intake increased the population of *Bacteroidales*, while the populations of *Clostridiales*, *Rhodospirillales*, and *Lactobacillales* decreased. At the family level, the populations of *Prevotellaceae*, *Peptostreptococcaceae*, *Bacteroidaceae*, *Rhodospirillaceae*, and *Erysipelotrichaceae* were reduced 1.7-fold, 5.8-fold, 2.7-fold, 3.5-fold, and 2.8-fold, respectively, in the RH group compared to the control group. In contrast, the population of *Lachnospiraceae* increased 1.6-fold.

### 3.3. Metabolomic Analysis, SCFAs, and the Ratios of NAD^+^/NADH and Nitrite/Nitrate

**Figure 2:**
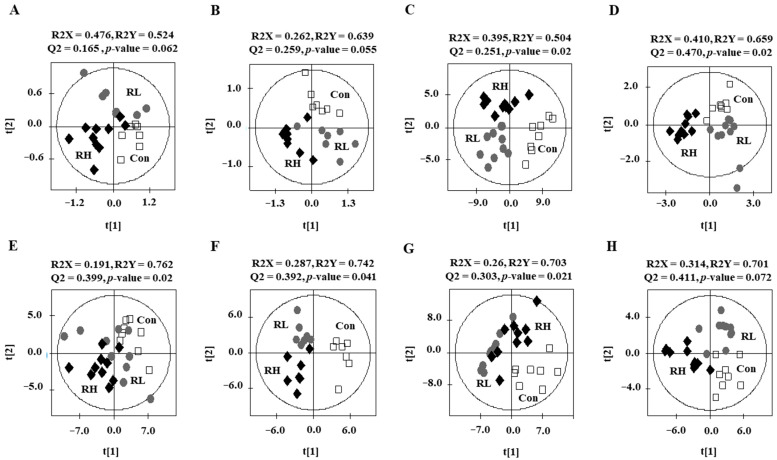
Partial least squares discriminant analysis (PLS-DA) score plots of metabolomic profiles from rats fed a control diet, low-dose RGE (RL; 100 mg RGE/kg body weight), or high-dose RGE (RH; 200 mg RGE/kg body weight). Score plots (**A**–**D**) show GC-MS data for (**A**) plasma, (**B**) liver, (**C**) kidney, and (**D**) intestinal content; score plots (**E**–**H**) show LC-MS data for the same sample types. The PLS-DA model performance was evaluated using R^2^X, R^2^Y, Q^2^, and permutation test *p*-values.

The metabolite profiles of the plasma, liver, kidney, and LIC from rats were analyzed using UPLC-Q-TOF MS (Figure S1) and GC-MS (Figure S2). The partial least squares discriminant analysis (PLS-DA) score plots showed that the control and RGE groups were significantly separated under statistically acceptable parameters (Figure 2). The quality parameters of the PLS-DA models for LC-MS and for GC-MS indicated that R^2^X, R^2^Y, and Q^2^ were more than 0.19, 0.70, and 0.30, respectively, and more than 0.26, 0.50, and 0.25, respectively. In addition, cross-validation with the permutation test for LC-MS and GC-MS indicated that the y-intercept values of R^2^ were <0.6 and those of Q^2^ were <−0.18 (Figure S3).

To identify metabolites contributing to the separations, all detected metabolites were statistically analyzed. A total of 42 plasma, 40 liver, 112 kidney, and 82 LIC metabolites, including fragments, detected by LC-MS and GC-MS were statistically affected by RGE. Among these, 17 plasma, 11 liver, 41 kidney, and 17 LIC metabolites were identified (Tables S2 and S3).

**Figure 3:**
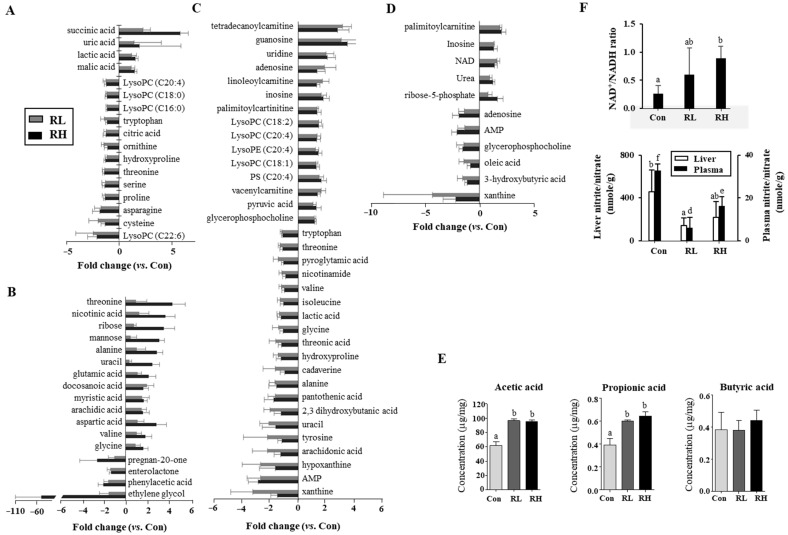
Fold changes for identified metabolites in (**A**) plasma, (**B**) intestinal contents, (**C**) kidney, and (**D**) liver in red ginseng (RG)-fed rats compared with controls. (**E**) Concentrations of short-chain fatty acids (SCFAs) in large intestinal contents. (**F**) Hepatic NAD^+^/NADH ratio and nitrite/nitrate levels in liver and plasma. Control; RL, low-dose RGE (100 mg RGE/kg body weight); RH, high-dose RGE (200 mg RGE/kg body weight). Data represented as mean ± SD. Bars with different letters are significantly different (*p* < 0.05).

In the plasma, the levels of lactic acid, succinic acid, malic acid, and uric acid were significantly increased in the RL and RH groups compared to the control group. Notably, succinic acid showed a more than five-fold increase. In contrast, the levels of proline, serine, threonine, hydroxyproline, cysteine, asparagine, ornithine, citric acid, tryptophan, and lysophosphatidylcholines (LPCs; C16:0, C18:0, C20:4, and C22:6) were significantly decreased (Figure 3A). In the LIC, the levels of threonine, nicotinic acid, ribose, mannose, uracil, glutamic acid, and aspartic acid were significantly increased—more than two-fold—in the RH group compared to the controls. In contrast, ethylene glycol showed a dramatic reduction of over 50-fold, while the pregnan-20-one and phenylacetic acid levels were reduced more than two-fold. Additionally, significant increases were observed in myristic acid, arachidic acid, glycine, valine, and docosanoic acid, whereas enterolactone was markedly decreased (Figure 3B). In the kidneys, the levels of pyruvic acid, uridine, inosine, guanosine, adenosine, glycerophosphocholine, carnitine, lysophosphatidylethanolamine (LPE, C20:4), PS (C20:4), and LPCs (C20:4, C18:1, and C18:2) were significantly increased in the RL and RH groups compared to the control group. In contrast, the levels of lactic acid, glycine, uracil, 2,3-dihydroxybutanoic acid, threonine, alanine, pyroglutamic acid, hydroxyproline, threonic acid, hypoxanthine, tyrosine, tryptophan, pantothenic acid, xanthine, arachidonic acid, isoleucine, valine, nicotinamide, cadaverine, and AMP were significantly decreased (Figure 3C). In the liver, the levels of inosine, NAD^+^, urea, ribose-5-phosphate, and palmitoylcarnitine were significantly increased in the RL and RH groups compared to the control group, whereas the levels of 3-hydroxybutyric acid, xanthine, glycerophosphocholine, adenosine, oleic acid, and AMP were significantly decreased (Figure 3D).

RGE intake also altered the levels of SCFAs (Figure 3E). The levels of acetic acid and propionic acid were significantly increased in the RL and RH groups, showing approximately 1.5-fold increases compared to the control group. RGE intake also altered the ratios of NAD^+^/NADH in the liver and nitrite/nitrate in the plasma and liver (Figure 3F). The NAD^+^/NADH ratios in the RL and RH groups were 2.3-fold and 3.5-fold higher, respectively, than those in the control group, whereas the nitrite/nitrate ratios in the plasma and liver were more than three-fold and two-fold lower, respectively, than those in the control group.

### 3.4. Metabolomic Pathway

**Figure 4:**
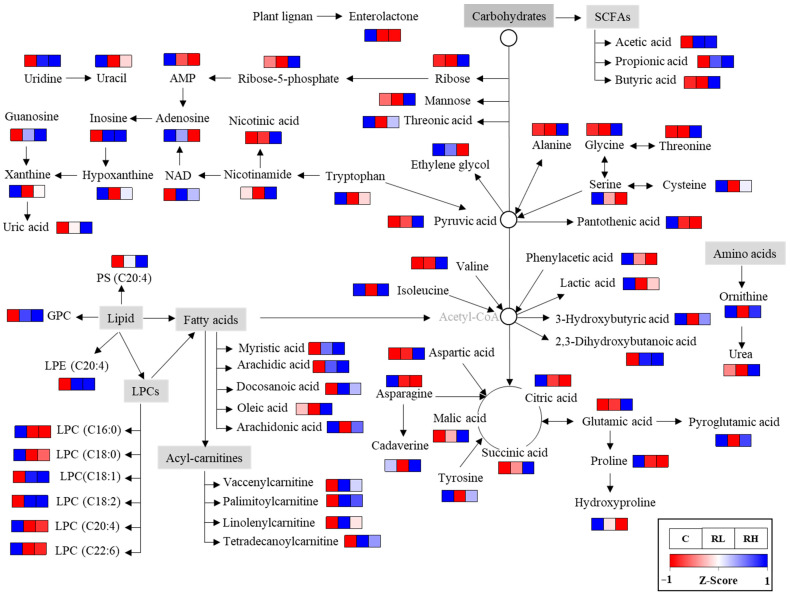
The proposed metabolomic pathway altered by red ginseng extract (RGE) intake based on the identified metabolites and their relative abundance. Control; RL, low-dose RGE (100 mg RGE/kg body weight); RH, high-dose RGE (200 mg RGE/kg body weight). Adjacent colored squares indicate Z-score-normalized levels of metabolites (red = upregulated; blue = downregulated).

Based on the discovered metabolites, an RGE-affected metabolomic pathway was proposed (Figure 4). RGE intake influenced multiple metabolic pathways, including those related to lipids, amino acids, sugars, nucleotides, the citric acid cycle, and SCFA metabolism. In particular, most LPCs were decreased, while the levels of other phospholipids, fatty acids, and acyl-carnitines were elevated. In addition, increases were observed in most amino acids, nucleotide degradation products, and acidic metabolites associated with energy production.

### 3.5. Effects of RGE on IgA and IL-12

**Figure 5:**
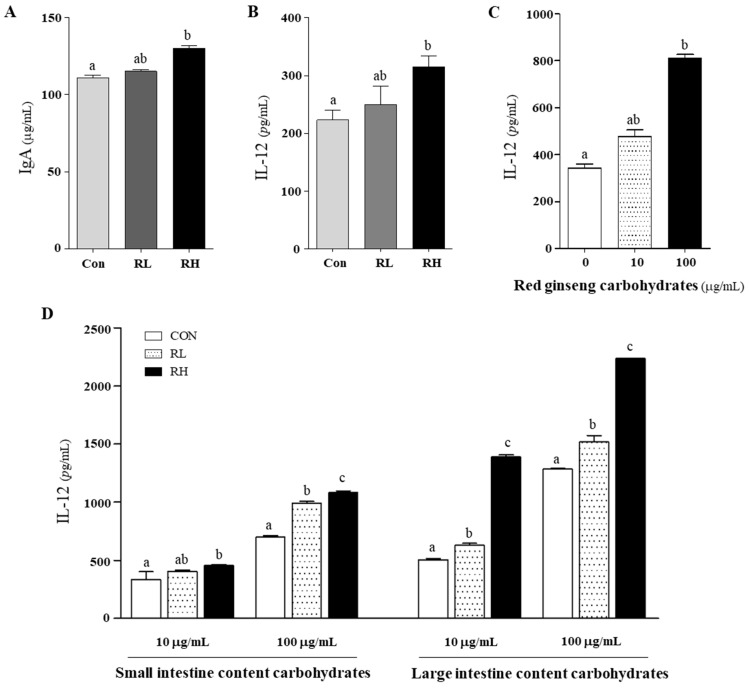
Immunomodulatory effects of red ginseng extract (RGE) on IgA and IL-12 production. (**A**) Blood IgA content and (**B**) IL-12 secretion by splenocytes isolated from rats from control group (fed normal diet), low-dose RGE (RL; 100 mg RGE/kg body weight) group, and high-dose RGE (RH; 200 mg RGE/kg body weight) group. (**C**) IL-12 production by naive rat splenocytes treated with RGE-derived carbohydrate fractions (RGE-C); (**D**) carbohydrates in small intestinal contents (SIC) and large intestinal contents (LIC) of control, RL, and RH rats. Data represented as mean ± SD. Bars with different letters are significantly different (*p* < 0.05).

The immunomodulatory effects of RGE on serum IgA levels and IL-12 cytokine secretion from splenocytes were evaluated (Figure 5). RGE intake resulted in a concentration-dependent increase in IgA levels and IL-12 production in splenocytes. Specifically, the serum IgA level in the RH group was 17% higher than that of the control group (Figure 5A), while IL-12 secretion from splenocytes isolated from the RH group was 41% higher than that of the control group (Figure 5B). Furthermore, the effects of RGE-derived carbohydrate fractions, including those isolated from raw RGE and those recovered from the small and large intestinal contents after RGE intake, on IL-12 production were analyzed using splenocytes isolated from control rats. The carbohydrate fractions isolated from raw RGE increased IL-12 production in a concentration-dependent manner, resulting in a 2.4-fold increase at 100 μg/mL compared to the untreated group (Figure 5C). Similarly, IL-12 production was elevated in splenocytes treated with carbohydrates derived from the SIC and LIC of RGE-fed rats compared to those from non-RGE-fed control rats (Figure 5D). At 10 μg/mL and 100 μg/mL, LIC-derived carbohydrate fractions from the RH group showed approximately 2.8-fold and 1.7-fold increases, respectively, compared to the control LIC group. In the SIC group, carbohydrate fractions from the RL group induced 1.3-fold and 1.5-fold increases at the same concentrations.

### 3.6. Correlation Analysis

The correlation analysis revealed distinct associations between host metabolites and physiological parameters (Figure 5). Liver, plasma, and kidney metabolites exhibited notable correlations with markers related to energy metabolism and blood biochemical parameters. In the liver, the NAD^+^/NADH ratio and HDL levels were positively correlated with inosine (*r* = 0.89), palmitoyl carnitine (*r* = 0.81), and NAD^+^ (*r* = 0.67). In the plasma, succinic acid (*r* = 0.95), malic acid (*r* = 0.97), and lactic acid (*r* = 0.97) showed strong positive correlations. In the kidneys, positive correlations were observed with lipid-derived metabolites (*r* > 0.43), nucleotide metabolites (*r* > 0.92), and pyruvic acid (*r* = 0.90). In contrast, most other metabolites showed negative correlations with the same parameters. However, contrasting correlation trends were observed for ALT, AST, TG, and nitrite. Strong correlations were also observed between the gut microbiota and gut-related parameters, including gut metabolites, SCFAs, and immune markers. *Peptostreptococcaceae*, *Rhodospirillaceae*, *Bacteroidaceae*, and *Erysipelotrichaceae* exhibited strong positive correlations with phenyl lactic acid (*r* > 0.90), enterolactone (*r* > 0.99), and ethylene glycol (*r* > 0.54). *Prevotellaceae* was positively correlated with ethylene glycol (*r* = 0.90) and pregnan-20-one (*r* = 1.00). In contrast, these bacterial taxa were negatively associated with SCFAs (−1.00 < *r* < −0.27), immune markers (−0.99 < *r* < −0.50), and most other gut metabolites, especially lipid metabolites (−1.00 < *r* < −0.44), including docosanoic acid (except in *Prevotellaceae*), myristic acid, and arachidic acid. However, *Lachnospiraceae* showed the opposite correlation trends.

## 4. Discussion

RGE intake significantly altered the gut microbiota composition, immune responses, SCFA production, and global metabolite profiles in healthy rats. In this healthy rat model, body weight was not significantly affected by RGE intake. However, the metabolic effects of RGE were not uniformly favorable, because LDL cholesterol was significantly increased despite reductions in TG, ALT, AST, and epididymal adipose tissue weight. This unexpected increase in LDL cholesterol may reflect altered hepatic cholesterol handling, including LDL receptor-mediated cholesterol clearance, HMG-CoA reductase-dependent cholesterol synthesis, or bile acid metabolism. However, these pathways were not directly assessed in the present study and require targeted mechanistic validation. Our findings align in part with previous clinical and animal studies demonstrating the beneficial effects of RG or ginseng intake on obesity and related metabolic disorders [21,22].

### 4.1. Gut Microbiota and Metabolites

RGE intake decreased opportunistic pathogenic bacteria (*Prevotellaceae*, *Peptostreptococcaceae*, *Bacteroidaceae*, *Rhodospirillaceae*, and *Erysipelotrichaceae*) and increased beneficial bacteria such as *Lachnospiraceae* (Figure 1), promoting gut homeostasis. These pathogenic bacteria contribute to inflammation, metabolic disorders, and gut barrier dysfunction, whereas *Lachnospiraceae* support SCFA production, metabolism, fiber fermentation, and antimicrobial peptide regulation, enhancing barrier integrity, immune balance, and pathogen resistance [23].

**Figure 6:**
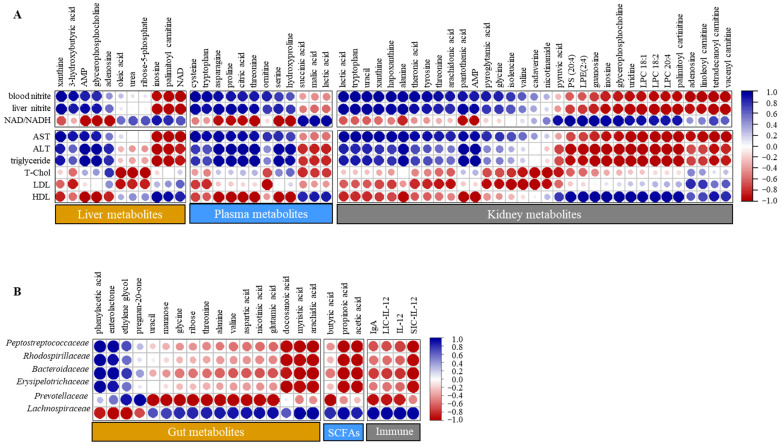
Correlation analysis among biochemical characteristics, gut microbiota, SCFAs, metabolites, and immune markers. (**A**) Correlations among liver, plasma, and kidney metabolites and biochemical parameters. (**B**) Correlations between gut microbiota and gut-related metabolites and immune markers. Circle color indicates correlation direction (blue, positive; red, negative), and circle size reflects correlation strength.

Consistent with microbial changes, RGE intake influenced SCFA production (Figure 3E), which supports energy metabolism and helps to prevent colon-associated diseases [24]. SCFAs also have therapeutic potential in ulcerative colitis, Crohn’s disease, and antibiotic-associated diarrhea [25,26]. Although this study did not directly investigate the relationships between RGE components and SCFA production, RGE-derived non-digestible carbohydrate components may serve as substrates for SCFA-producing *Lachnospiraceae*. Indeed, the correlation analysis showed that *Lachnospiraceae* were positively correlated with SCFAs and immune markers (IgA and IL-12), whereas pathogenic taxa were reduced by RGE, with a negative correlation (Figure 6).

The metabolomic profiles of the colonic contents revealed the accumulation of free amino acids (threonine, alanine, glutamic acid, aspartic acid, valine, and glycine), monosaccharides (mannose and ribose), free fatty acids (docosanoic acid, myristic acid, and arachidic acid), and nucleoside derivatives (nicotinic acid and uracil). These shifts suggest enhanced mucin degradation, potentially driven by mucin-degrading bacteria such as members of the *Lachnospiraceae* family, which are promoted by RGE intake [27]. For instance, threonine, a major mucin component, provides substrates for microbial growth and exposes O-glycan residues that facilitate colonization [28]. Conversely, RGE intake reduced the colonic levels of pregnan-20-one, phenylacetic acid, and ethylene glycol, which have been linked to physiological dysfunction or toxicity [29,30,31]. Phenylacetic acid, a bacterial breakdown product of amino acids, is associated with hepatic fat accumulation in non-diabetic obese women [32], while pregnan-20-one is linked to depression and the inhibition of GABA production [33]. Interestingly, ethylene glycol, which is associated with central nervous system dysfunction and renal toxicity [34], was significantly reduced by RGE intake.

### 4.2. Metabolite Profiles of Blood, Liver, and Kidneys and Nitrite/Nitrate Levels

RGE intake affected the metabolite profiles of the blood, kidneys, and liver (Figure 3). Succinic acid, uric acid, lactic acid, and malic acid were among the acidic compounds in the blood that increased with RGE intake. This could lead to moderate acidosis, which relaxes arteriolar smooth muscle, boosting microvascular blood flow and decreasing vascular resistance [35,36]. However, the marked increase in succinic acid should be interpreted cautiously, because succinate can also act as a signaling molecule and may reflect altered mitochondrial efficiency or gut-derived signaling. As mitochondrial function and succinate-related signaling were not directly assessed in this study, this hypothesis requires further validation. Moreover, in prehypertensive subjects, RG consumption lowered blood pressure in association with decreases in lipoprotein-associated phospholipase A2 activity and LPCs [37]. Consistently, our study showed that RGE reduced plasma LPCs (16:0, 18:0, 20:4, and 22:6), which are known to act as inflammatory stimuli, function as secondary messengers, and contribute to vasodilation [38]. Together, these findings suggest that RGE intake may improve vascular health through both pH-mediated vasodilation and the modulation of pro-inflammatory lipids.

In the kidneys, RGE intake led to the accumulation of lipid metabolites, including LPCs, acyl-carnitines, and glycerolphosphocholine, which may contribute to kidney dysfunction [39,40]. Conversely, increased purine nucleosides may enhance vascular regulation, anti-inflammatory responses, and cell survival mechanisms, thereby strengthening the capacity of the kidneys for tissue repair [41]. Moreover, RGE intake reduced the levels of amino acids, AMP, xanthine, and hypoxanthine, easing proteolytic strain and limiting xanthine oxidase-driven H_2_O_2_ production, thereby reducing oxidative stress [42].

In the liver, the decrease in AMP along with increases in NAD^+^ and the NAD^+^/NADH ratio indicate a high-energy state with enhanced oxidative metabolism. Reduced AMP suppresses AMPK activity, downregulating catabolic pathways, while a high NAD^+^/NADH ratio signals active dehydrogenases in the TCA cycle and β-oxidation, boosting ATP production [43]. These findings support previous reports that ginseng and its ginsenosides enhance energy expenditure and activate β-oxidation. Additionally, RGE intake decreased nitrite and nitrate levels in both the liver and blood, suggesting that the antioxidant activity of RGE [44] reduces reactive oxygen species-mediated NO oxidation and may directly inhibit nitric oxide synthase activity, lowering NO synthesis. The vasodilatory effects of RGE improve blood flow and reduce compensatory NO demands [45], while anti-inflammatory effects may further limit NO production. Together, these antioxidant, anti-inflammatory, and vascular effects demonstrate the capacity of RGE to modulate NO metabolism.

The correlation analysis further supported the observed metabolic changes (Figure 6). In the liver, a higher NAD^+^/NADH ratio was positively correlated with NAD^+^ and palmitoylcarnitine, suggesting enhanced oxidative metabolism. In contrast, negative correlations with AMP and xanthine suggest reduced energy stress and oxidative burdens. In the blood, succinic and malic acids also showed positive correlations with the hepatic NAD^+^/NADH ratio, reflecting active energy metabolism. Additionally, nitrite levels in both the liver and blood were negatively correlated with the NAD^+^/NADH ratio, suggesting that reduced nitrite/nitrate levels after RGE intake may be linked to improved metabolic efficiency and lower oxidative stress [46]. These findings suggest that RGE intake contributes to enhanced energy balance and redox homeostasis in both hepatic and systemic metabolisms.

### 4.3. Immune Responses

RGE intake influenced immune responses in rats by elevating serum IgA levels and splenic IL-12 production (Figure 5A,B). Within the mucosal immune system, IgA plays a key role in maintaining intestinal homeostasis, regulating the microbiota composition via secretory IgA, and providing defense against toxins and pathogens [47,48]. In this study, RGE intake significantly increased the serum IgA levels, suggesting a potential role in mucosal immune enhancement and gut microbiota homeostasis. Additionally, IL-12, a key cytokine that activates natural killer cells and T lymphocytes in the cellular immune system [49], was increased in the spleens of RGE-fed rats compared with controls (Figure 5B). Furthermore, the ex vivo treatment of splenocytes with either intact RGE carbohydrate fractions (Figure 5C) or carbohydrate residues isolated from the small and large intestinal contents (SIC and LIC) of RGE-fed rats significantly increased IL-12 production compared with that in the control group (Figure 5D). Notably, LIC-derived carbohydrate residues elicited a greater IL-12 response than either the intact RGE carbohydrate fractions or the SIC-derived residues. These findings indicate that partially digested RGE carbohydrate residues stimulate IL-12 production more effectively than intact RGE carbohydrate fractions. Although the underlying mechanisms remain unclear, previous studies have reported that RGE-derived polysaccharides enhance both IgA and IL-12 responses [47]. In mice, oral administration and intraperitoneal injection increased mucosal and systemic IgA [50], and RGE polysaccharide treatment enhanced IL-12 production and NK/T-cell activation in vitro [51].

## 5. Conclusions

In conclusion, RGE intake in healthy rats modulated the gut, systemic metabolism, and immune function. Intestinally, RGE reshaped the microbiota population by depleting opportunistic pro-inflammatory taxa and enriching SCFA-producing *Lachnospiraceae*, alongside elevated SCFA levels. Systemically, RGE intake altered the plasma, hepatic, and renal metabolite profiles, suggesting changes in lipid, purine, and energy metabolism, as well as liver-related biochemical parameters. Interestingly, partially digested RGE-derived carbohydrate residues in the gut after RGE intake amplified IL-12 production, highlighting a gut–immune axis effect. However, the carbohydrate fractions used in this study were not chemically characterized in detail. In addition, this study did not validate all affected metabolites, gut microbiota, and biochemical parameters, and the small sample size should be considered when interpreting the multi-omics integration and correlation analyses. Further research in disease models is needed to confirm our findings and clarify the underlying mechanisms. Despite these limitations, these findings suggest a role for RGE in metabolic and immune modulation under normal physiological conditions. However, because LDL cholesterol was increased in the RGE-treated groups, further studies are needed to clarify the effects of REG on cholesterol metabolism and its cardiovascular relevance.

## Figures and Tables

**Table 1:** General characteristics of rats fed a normal diet with or without red ginseng extract (RGE).

	Control	RL	RH	*p*-Value
Body weight gain (g)	4.79 ± 0.38	4.54 ± 0.57	4.55 ± 0.52	0.53
Food intake (g/day)	23.17 ± 1.79	22.37 ± 1.39	22.12 ± 1.49	0.15
Epididymal adipose tissue (g)	3.24 ± 0.21 ^b^	2.81 ± 0.50 ^ab^	2.69 ± 0.61 ^a^	0.09
Liver (g)	9.38 ± 0.96	9.17 ± 0.98	9.57 ± 1.18	0.70
Blood TG (mg/dL)	26.00 ± 3.16 ^b^	18.50 ± 6.14 ^a^	17.50 ± 3.87 ^a^	0.05
Blood TC (mg/dL)	85.25 ± 14.57	95.20 ± 10.71	91.20 ± 4.82	0.39
LDL cholesterol (mg/dL)	20.50 ± 6.24 ^a^	28.80 ± 3.70 ^b^	29.80 ± 3.56 ^b^	0.02
HDL cholesterol (mg/dL)	77.00 ± 9.42	83.40 ± 9.32	81.00 ± 5.92	0.53
ALT (U/L)	32.00 ± 7.64 ^b^	27.00 ± 3.46 ^a^	28.60 ± 1.67 ^ab^	0.05
AST (U/L)	95.00 ± 3.00 ^c^	66.00 ± 2.83 ^a^	72.25 ± 3.50 ^b^	1.45 × 10^−6^

RL, low-dose red ginseng extract (100 mg/kg); RH, high-dose red ginseng extract (200 mg/kg); TG, triglycerides; TC, total cholesterol; ALT, alanine aminotransferase; AST, aspartate aminotransferase. Values are expressed as mean ± SD (n = 5), and different letters within the same row indicate significant differences at *p* < 0.05.

## Data Availability

The datasets generated and analyzed during the current study are not publicly available due to the sensitivity of personal data but are available from the corresponding author on reasonable request.

## Supplementary Materials

The following supporting information can be downloaded at https://www.mdpi.com/article/10.3390/nu18091462/s1, Table S1: Composition analysis of red ginseng extract; Table S2: Plasma, liver, kidney and intestinal contents metabolites from red ginseng-administrated-rat analyzed using LC-MS; Table S3: Plasma, liver, kidney and intestinal content metabolites from red ginseng-administrated-rat analyzed using GC-MS; Figure S1: Presentative chromatograms of (A) Plasma, (B) Liver, (C) Kidney, and (D) Intestincal content using LC/MS; Figure S2: Presentative chromatograms of (A) Plasma, (B) Liver, (C) Kidney, and (D) Intestinal content using GC/MS; Figure S3: A permutation test was performed to validate the PLS-DA model of (A,E) Plasma, (B,F) Liver, (C,G) Kidney, and (D,H) intestinal contents of rats with or without fed red ginseng of GC-MS and LC-MS, respectively; Figure S4: Rarefaction curves of OTUs defined by sequence variation in the fecal. The X-axis shows the number of sequences in each sample, while the Y-axis shows the numbers of operational taxonomic units (OTU) encountered; Figure S5: Correlation analysis (A) Correlations among liver, plasma, and kidney metabolites and biochemical parameters. (B) Correlations between gut microbiota and gut-related metabolites and immune markers.
