# Supplementary material for: Red Ginseng Extract Intake and Changes in Metabolite Profiles, Gut Microbiota, and Immune Responses of Healthy Rats
Source: Nutrients. 2026 May 2;18(9):1462. doi: 10.3390/nu18091462 (PMC13164613; doi:10.3390/nu18091462)
Supplement: Supplementary file 1 [file nutrients-18-01462-s001.zip › nutrients-4271209-supplementary.pdf]

Table S1. Composition analysis of red ginseng extract

**Parameters**	
Brix (°Bx)	45.60
Yield (%)	24.95
Total sugar (%)	70.07 ±2.42
Acidic polysaccharide (%)	26.84 ± 0.43
Total phenolic compound (%)	0.78 ± 0.04
Total ginsenosides (mg/g dry extract)	29.69
Solid content (mg/g)	480.50 ± 10.70

Table S2. Plasma, liver, kidney and intestinal contents metabolites from red ginseng-administrated-rat analyzed using LC-MS

Sample	Compound	Observed mass (M+H)	Actual mass (M+H)	Mass error (mDa)	Fragment	*p*-value	VIP
Plasma	Uric acid	169.036	169.0362	-2	141, 126	3.90e-02	1.27
	Lysopc (C16:0)	496.3408	496.3403	-5	184, 104	7.34e-02	1.18
	Lysopc (C18:0)	524.3716	524.3716	0	506, 184, 104	3.10e-02	1.40
	Lysopc (C20:4)	544.3402	544.3397	5	184, 104	4.69e-02	1.28
	Lysopc (C22:6)	568.3392	568.3403	-11	550, 184, 104	1.44e-04	1.92
Liver	Glycerophosphocholine	258.1112	258.1106	6	125, 104	4.43e-02	1.29
	Adenosine	268.1045	268.1046	-1	136	8.56e-04	1.73
	Inosine	269.0885	269.0886	-1	137	4.39e-02	1.30
	Amp	348.0707	348.0709	-2	136	4.97e-03	1.60
	Nad	664.1159	664.1169	-10	428, 348, 136	2.74e-04	1.86
	Palmitoylcarnitine	400.3421	400.3427	-6	85	3.28e-02	1.35
Kidney	Hypoxanthine	137.0465	137.0471	-6	136	5.33e-03	1.40
	Xanthine	153.0414	153.0417	-3	136, 110, 82	1.18e-02	1.28
	Tyrosine	182.0813	182.0817	-4	165, 136, 123	3.34e-02	1.29
	Tryptophan	205.0979	205.0977	2	188, 146, 118	5.14e-02	1.02
	Pantothenic acid	220.1185	220.1185	0	202, 184, 124	2.42e-05	1.62
	Glycerophosphocholine	258.111	258.1106	4	125, 104	5.07e-03	1.25
	Adenosine	268.1038	268.1046	-8	136	2.39e-02	1.36
	Guanosine	284.0992	284.0995	-3	152	3.69e-05	1.57
	Amp	348.0706	348.0709	-3	136	1.07e-08	1.90
	Tetradecanoylcarnitine	372.3106	372.3114	-8	313, 211, 85	3.80e-04	1.42
	Linolenylcarnitine	424.3418	424.3427	-9	85	1.64e-04	1.72
	Palimitoylcarnitine	400.342	400.3427	-7	85	2.17e-03	1.26
	Vaccenylcarnitine	426.3576	426.3583	-7	85	7.04e-05	1.59
	Lysope (C20:4)	502.2926	502.292	6	361, 104	2.31e-03	1.61
	Lysopc (C20:4)	544.3396	544.3397	-1	184, 104	9.66e-03	1.12
	Lysopc (C18:1)	522.3555	522.356	-5	184, 104	1.80e-04	1.20

	Lysopc (C18:2)	520.3398	520.3391	7	184, 104	1.80e-04	1.43
	Ps (c20:4)	546.2822	546.2832	-10	184	5.46e-02	1.24
Intestinal content	Enterolactone	299.1279	299.1283	-4	133, 107	4.39e-02	1.29
	Pregnan-20-one, 17-(acetyloxy)-3-hydroxy-6-methyl-, (3b,5b,6a)-	391.2862	391.2848	14	373, 355	2.33e-03	1.30

Table S3. Plasma, liver, kidney and intestinal content metabolites from red ginseng-administrated-rat analyzed using GC-MS

Sample	Compound	RI	*p*-value	VIP	Identified
Plasma	Lactic acid	1052	1.82e-02	1.14	MS, RI, ST
	Proline	1292	4.22e-04	0.75	MS, RI, ST
	Succinic acid	1311	5.91e-03	3.71	MS, RI, ST
	Serine	1354	3.17e-03	1.44	MS, RI, ST
	Threonine	1377	1.38e-05	0.82	MS, RI, ST
	Malic acid	1485	9.07e-03	0.92	MS, RI, ST
	Hydroxyproline	1521	1.10e-03	0.53	MS, RI, ST
	Cysteine	1549	3.55e-02	1.77	MS, RI, ST
	Asparagine	1663	2.79e-02	1.78	MS, RI, ST
	Ornithine	1809	4.56e-02	1.27	MS, RI
	Citric acid	1813	5.72e-02	0.48	MS, RI, ST
	Tryptophan	2187	5.47e-02	1.02	MS, RI, ST
Intestinal Content	Ethylene glycol	990	1.51e-02	0.53	MS, RI, ST
	Valine	1089	5.45e-04	0.96	MS, RI, ST
	Alanine	1102	4.30e-06	1.84	MS, RI, ST
	Nicotinic acid	1289	5.63e-07	2.42	MS, RI
	Phenylacetic acid	1293	1.68e-06	0.89	MS, RI
	Glycine	1300	2.00e-02	0.96	MS, RI, ST
	Uracil	1329	3.16e-09	3.15	MS, RI
	Threonine	1373	8.73e-08	2.62	MS, RI, ST
	Aspartic acid	1515	1.25e-05	1.68	MS, RI, ST
	Glutamic acid	1616	9.17e-05	1.14	MS, RI, ST
	Ribose	1673	4.94e-08	2.42	MS, RI
	Myristic acid	1846	1.36e-09	3.37	MS, RI
	Mannose	1891	1.67e-03	3.37	MS, RI, ST
	Arachidic acid	2438	2.74e-02	0.97	MS, RI
	Docosanoic acid	2635	1.12e-03	1.27	MS, RI
Liver	3-hydroxybutyric acid	1157	6.12e-02	0.97	MS, RI
	Urea	1241	4.14e-02	0.73	MS, RI, ST
	Xanthine	2011	4.27e-02	2.9	MS, RI
	Ribose-5-phosphate	2106	1.75e-02	1.87	MS, RI
	Oleic acid	2213	3.70e-02	1.3	MS, RI
Kidney	Pyruvic acid	1046	5.01e-02	1.27	MS, RI
	Lactic acid	1056	9.15e-03	1.5	MS, RI, ST
	Glycine	1119	4.23e-02	1.3	MS, RI, ST
	Valine	1213	2.74e-02	1.45	MS, RI, ST
	Isoleucine	1291	3.09e-02	1.41	MS, RI

	Glycine	1302	7.48e-02	1.2	MS, RI, ST
	Uracil	1332	1.27e-03	1.73	MS, RI, ST
	2,3-dihydroxybutanoic acid	1338	1.09e-02	1.51	MS, RI
	Threonine	1377	1.19e-02	1.45	MS, RI, ST
	Alanine	1417	5.60e-02	1.15	MS, RI, ST
	Nicotinamide	1473	8.52e-03	1.39	MS, RI
	Pyroglutamic acid	1515	9.50e-03	1.55	MS, RI
	Hydroxyproline	1523	3.23e-02	1.36	MS, RI, ST
	Threonic acid	1540	1.35e-02	1.47	MS, RI
	Hypoxanthine	1799	3.69e-03	1.64	MS, RI
	Cadaverine	1848	1.91e-02	1.41	MS, RI
	Tyrosine	1879	1.21e-02	1.48	MS, RI, ST
	Pantothenic acid	1987	5.72e-03	1.57	MS, RI
	Xanthine	2013	2.44e-02	1.39	MS, RI
	Arachidonic acid	2365	1.08e-02	1.49	MS, RI
	Uridine	2440	2.96e-04	1.84	MS, RI, ST
	Inosine	2568	6.31e-04	1.72	MS, RI
	Guanosine	2766	2.55e-02	1.31	MS, RI

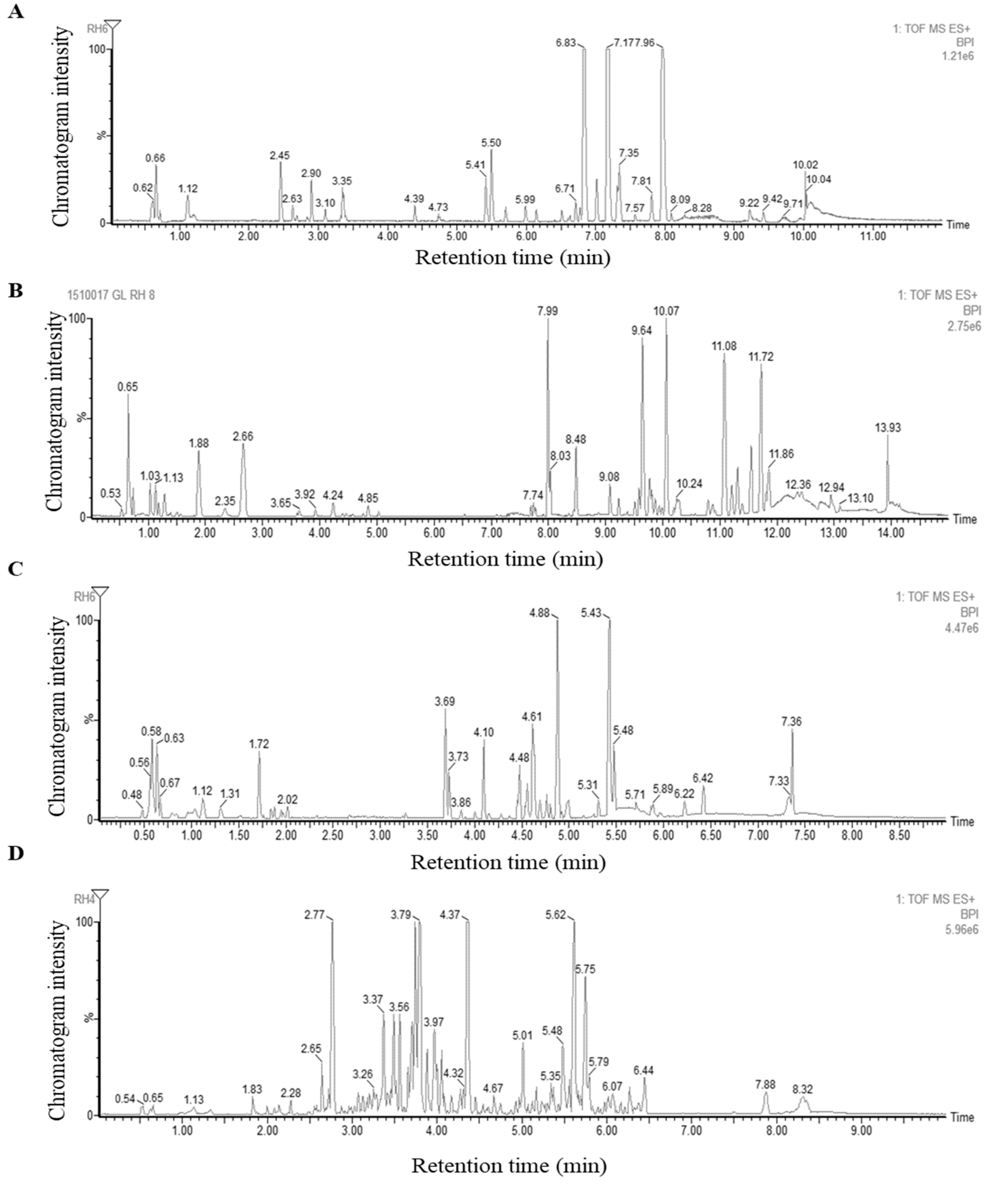

Fig. S1. Presentative chromatograms of **A.** Plasma, **B.** Liver, **C.** Kidney, and **D.** Intestincal content using LC/MS

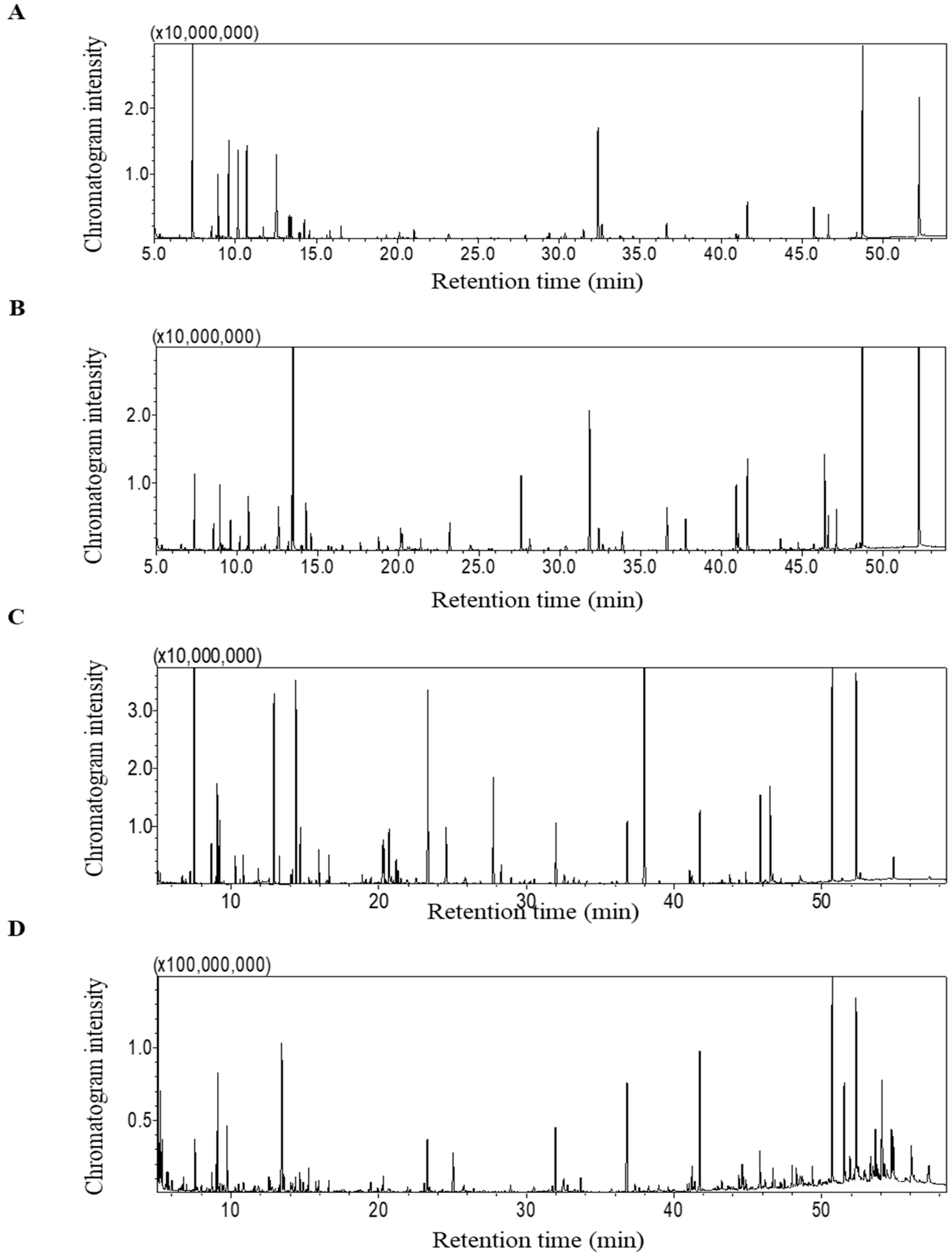

Fig. S2. Presentative chromatograms of **A.** Plasma, **B.** Liver, **C.** Kidney, and **D.** Intestinal content using GC/MS

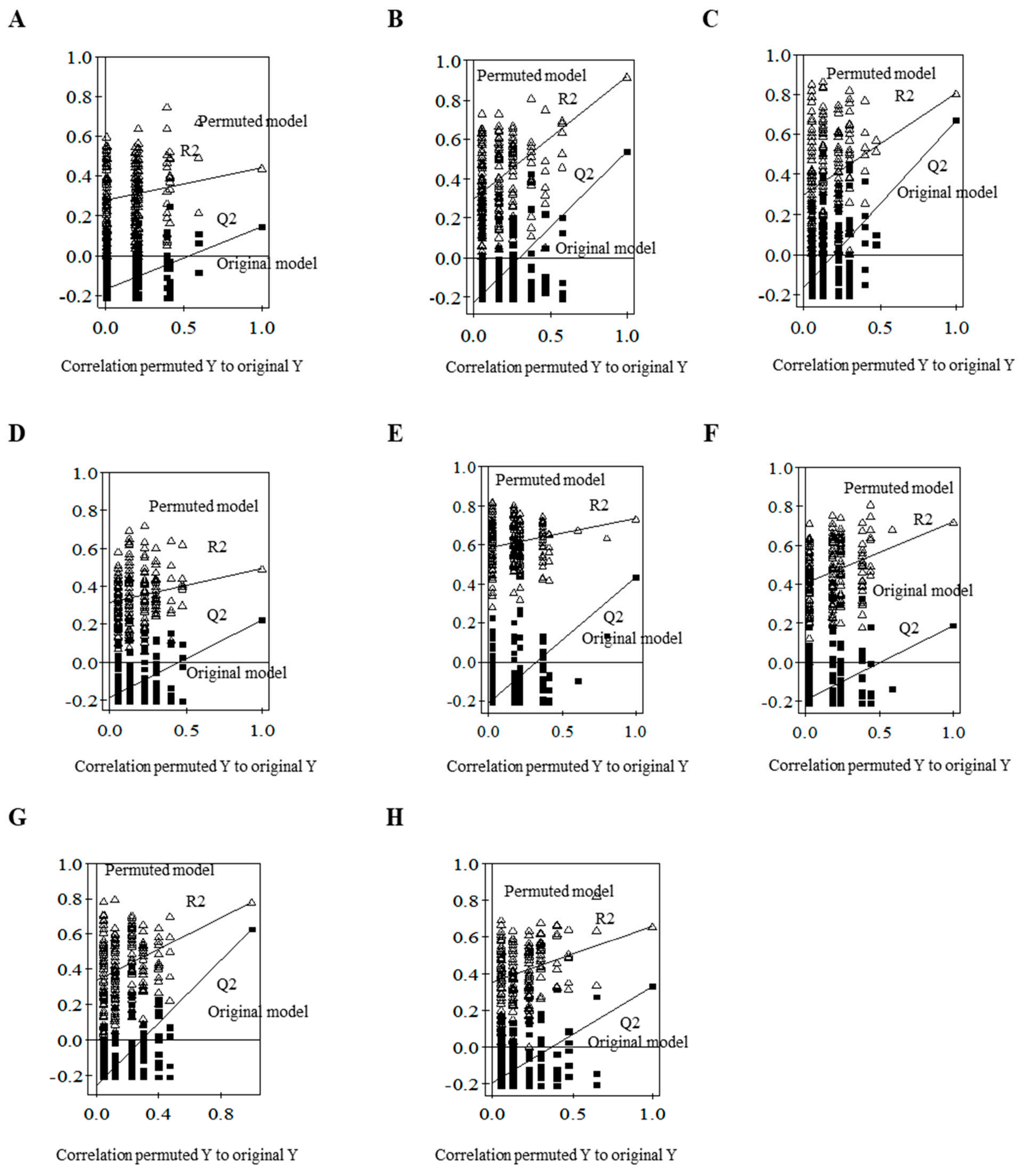

Fig. S3. A permutation test was performed to validate the PLS-DA model of **A, E.** Plasma, **B, F.** Liver, **C, G.** Kidney, and **D. H.** intestinal contents of rats with or without fed red ginseng of GC-MS and LC-MS, respectively.

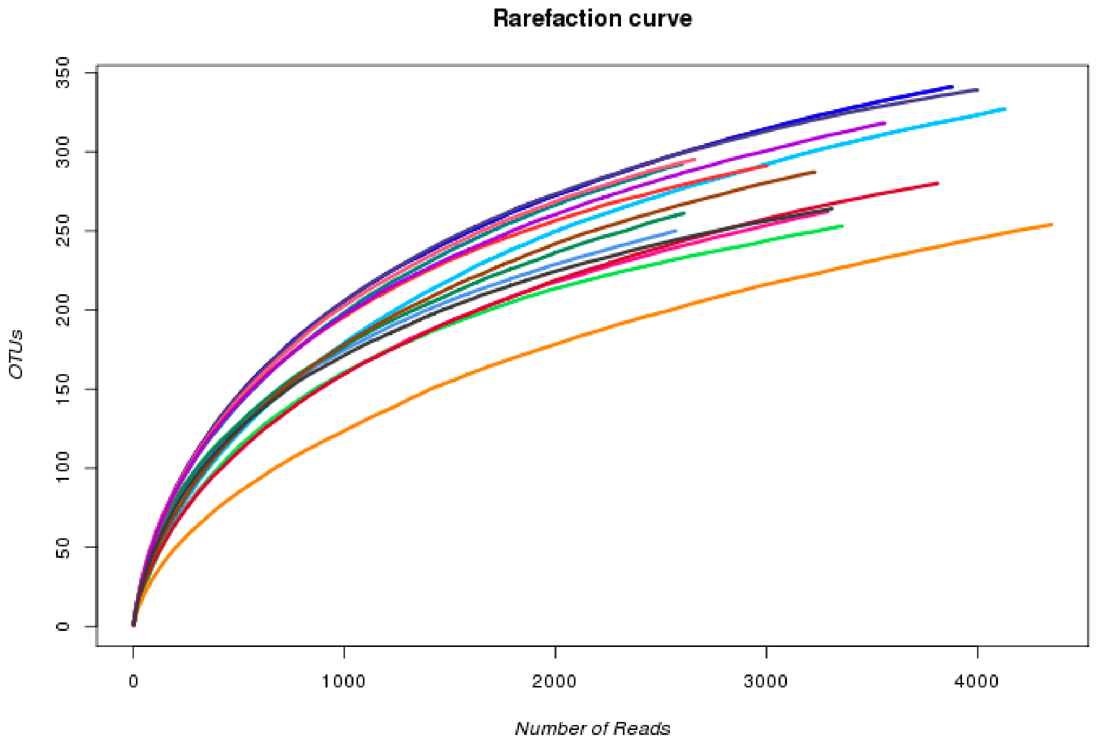

Fig. S4. Rarefaction curves of OTUs defined by sequence variation in the fecal. The X-axis shows the number of sequences in each sample, while the Y-axis shows the numbers of operational taxonomic units (OTU) encountered.

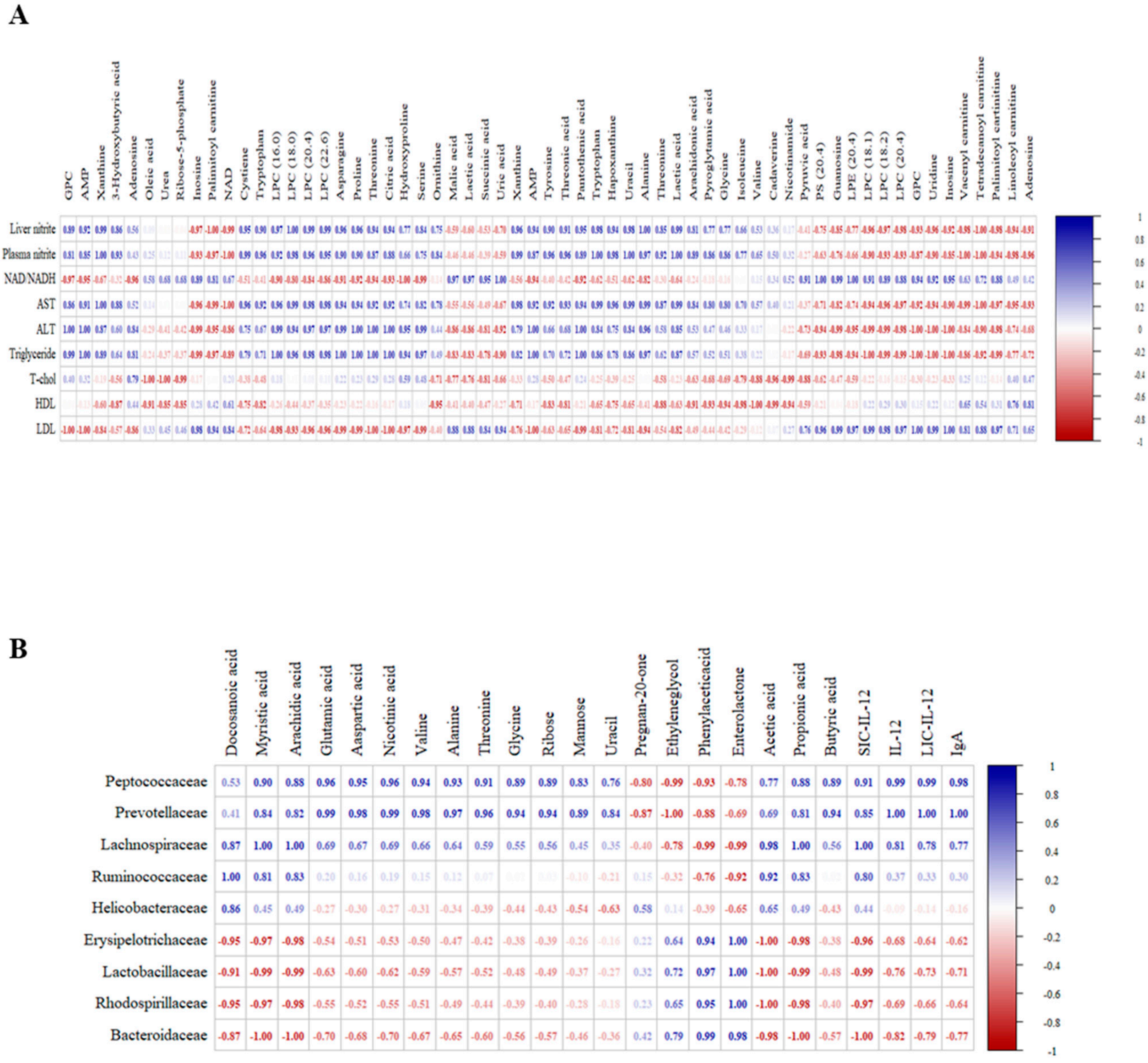

Fig. S5. Correlation analysis **A.** Correlations among liver, plasma, and kidney metabolites and biochemical parameters. **B.** Correlations between gut microbiota and gut-related metabolites and immune markers.
